# Supplementary material for: Chronotype-Dependent Sleep Loss Is Associated with a Lower Amplitude in Circadian Rhythm and a Higher Fragmentation of REM Sleep in Young Healthy Adults
Source: Brain Sci. 2023 Oct 19;13(10):1482. doi: 10.3390/brainsci13101482 (PMC10605513; doi:10.3390/brainsci13101482)
Supplement: Supplementary file 1 [file brainsci-13-01482-s001.zip › brainsci-2640560-supplementary.pdf]

Supplemental Figures

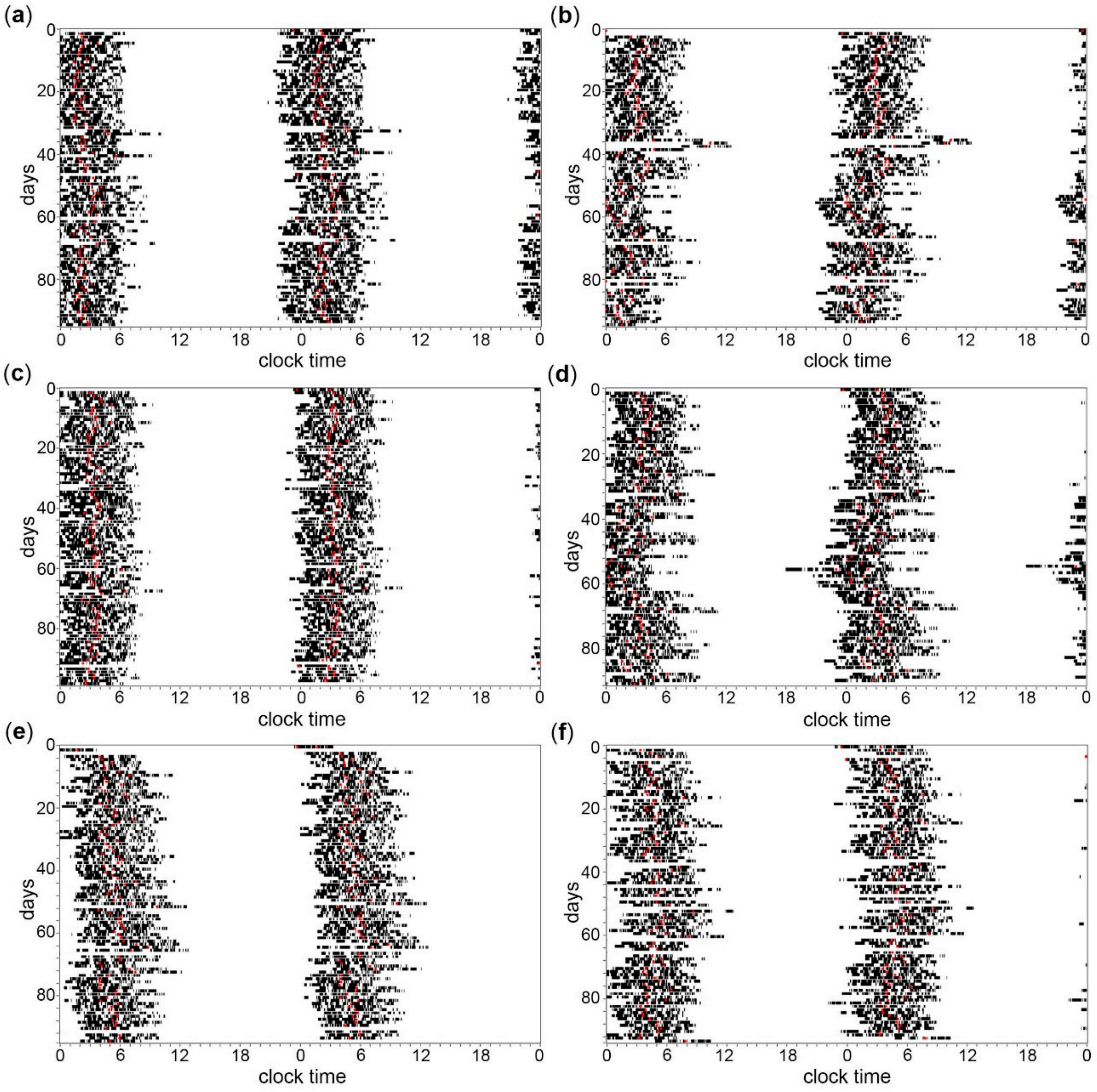

Figure S1. Longitudinal recordings of total sleep under real-life conditions. Representative actograms of total sleep of six different subjects. Each day is plotted twice. The sleep is shown as black bars, acrophases as red dots. The subjects vary in terms of their midpoint of sleep on free days, defining the chronotype. Subjects shown in (**a**) and (**b**) represent earlier chronotypes; subjects in (**c**) and (**d**) represent intermediate chronotypes; subjects in (**e**) and (**f**) represent later chronotypes. Chronotype was defined by the midpoint of sleep on free days (weekends) calculated based on sleep onset and sleep offset.

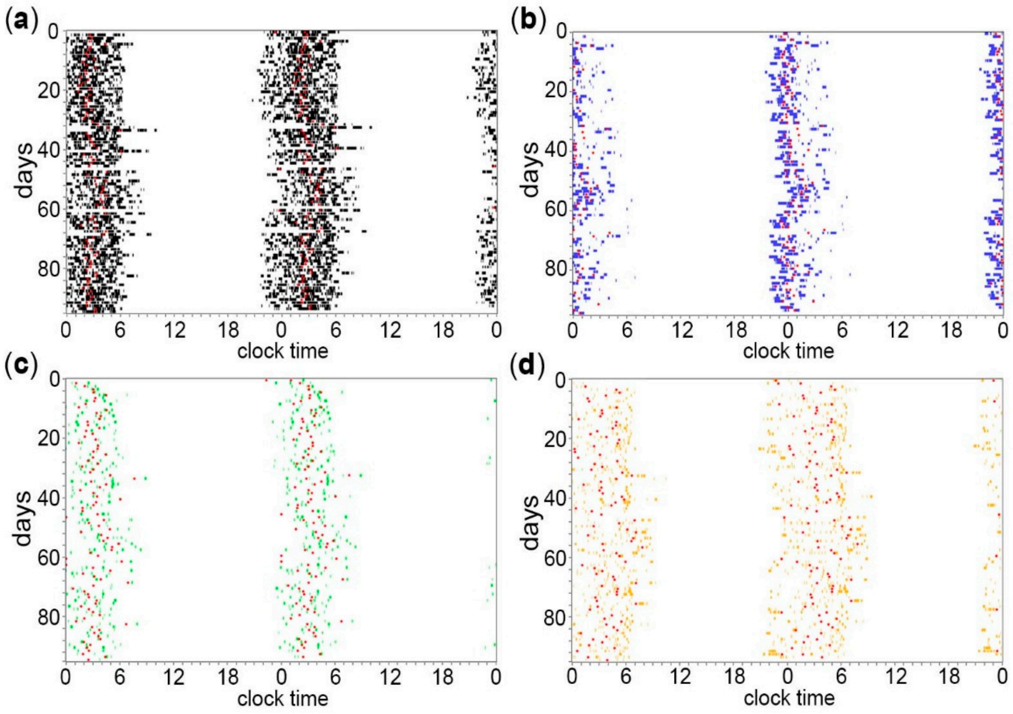

Figure S2. Longitudinal recordings of sleep stages under real-life conditions. Representative double-plotted actograms of sleep stages of one subject. (**a**) light sleep; (**b**) deep sleep; (**c**) REM sleep; (**d**) wake after sleep onset. Note that deep sleep episodes are earlier and more consolidated than those of REM sleep and wake after sleep onset.

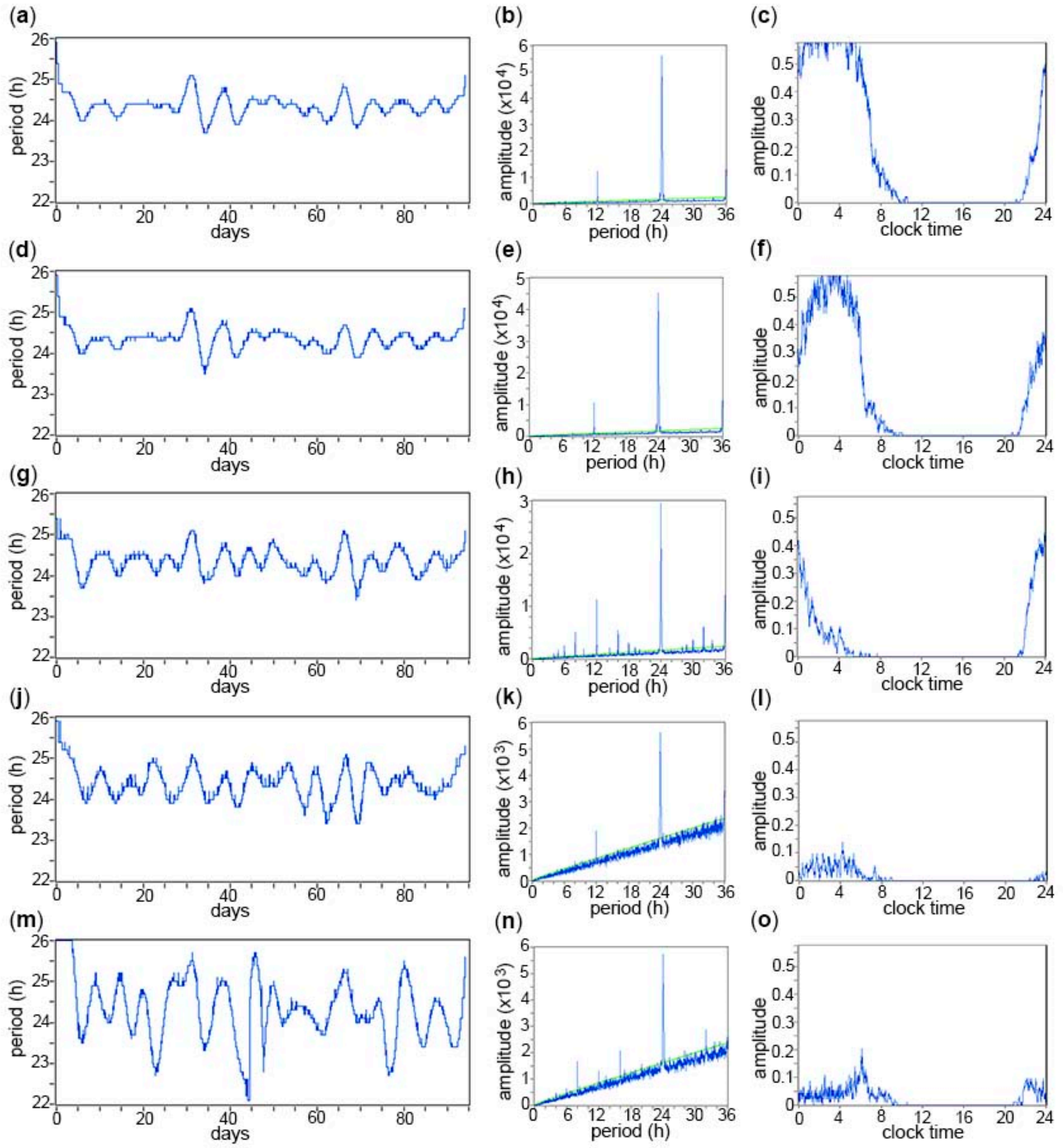

Figure S3. Representative analyses of circadian rhythms of total sleep and sleep stages of one subject. **a**, **b**, **c**) total sleep; (**d**, **e**, f) light sleep; (**g**, **h**, **i**) deep sleep; (**j**, **k**, **l**) REM sleep; (**m**, **n**, **o**) wake after sleep onset. (**a**, **d**, **g**,**j**, **m**) Morlet continuous wavelet transform analyses show day-to-day osciallations around the 24 h period, indicating the circadian nature of sleep and sleep stages. (**b**, **e**, **h**, **k**, **n**) Chi squared periodograms show the highest amplitudes in the 24 h period for total sleep and all sleep stages. The amplitude of the 24 h period is a measure of circadian rhythm stability. In addition, the 12 h period shows a high amplitude in total sleep, light sleep, deep sleep and REM sleep, indicating ultradian rhythms. (**c**, **f**, **i**, **l**, **o**) Activity profiles illustrate the phase and the 24 h amplitude of total sleep and sleep stages.
